# Vinculin and Rab5 Complex Is Requited for Uptake of *Staphyrococcus aureus* and Interleukin-6 Expression

**DOI:** 10.1371/journal.pone.0087373

**Published:** 2014-01-23

**Authors:** Makoto Hagiwara, Eitoyo Kokubu, Shinsuke Sugiura, Toshinori Komatsu, Hiroyuki Tada, Ryutaro Isoda, Naomi Tanigawa, Yoshiko Kato, Naoyuki Ishida, Kaoru Kobayashi, Misako Nakashima, Kazuyuki Ishihara, Kenji Matsushita

**Affiliations:** 1 Department of Oral Disease Research, National Center for Geriatrics and Gerontology, Obu, Aichi, Japan; 2 Department of Microbiology, Tokyo Dental College, Chiba, Japan; University of California Merced, United States of America

## Abstract

Vinculin, a 116-kDa membrane cytoskeletal protein, is an important molecule for cell adhesion; however, little is known about its other cellular functions. Here, we demonstrated that vinculin binds to Rab5 and is required for *Staphylococcus aureus* (*S. aureus*) uptake in cells. Viunculin directly bound to Rab5 and enhanced the activation of *S. aureus* uptake. Over-expression of active vinculin mutants enhanced *S. aureus* uptake, whereas over-expression of an inactive vinculin mutant decreased *S. aureus* uptake. Vinculin bound to Rab5 at the N-terminal region (1-258) of vinculin. Vinculin and Rab5 were involved in the *S. aureus*-induced phosphorylation of MAP kinases (p38, Erk, and JNK) and IL-6 expression. Finally, vinculin and Rab5 knockdown reduced infection of *S*. *aureus*, phosphorylation of MAPKs and IL-6 expression in murine lungs. Our results suggest that vinculin binds to Rab5 and that these two molecules cooperatively enhance bacterial infection and the inflammatory response.

## Introduction

Rab small GTPases are conserved membrane trafficking proteins in all eukaryotes, and they mediate various steps in membrane trafficking, including vesicle budding, vesicle movement, vesicle docking to specific membranes, and vesicle fusion [1]. Rab cycling between the GDP-bound (inactive) from and the GTP-bound (active) form is regulated by guanine nucleotide exchange factors (GEF), GTPase-activating proteins (GAPs), and GDP dissociation inhibitors [2]. This strict control is critical for the correct activation of Rab in time and space. More than 60 Rabs have been identified so far. Each Rab is localized to a specific membrane and controls a specific transport step [1], [3]. For example, Rab5 is localized to early endosomes and the plasma membrane, and it is essential for early stages of endocytosis and for fusion of the early endosome [4], [5]. Rab5 has been shown to be involved in the internalization of many extracellular materials such as nutrients [6], growth factors [7], [8], [9], viruses [10], [11], [12], [13] and bacterias [14], [15], [16], [17]. A large number of Rab5-interacting proteins including EEA1 [18], [19], Rabaptin-5 [20], [21], [22], phosphatidylinositol 3-kinases [23], [24], Rabankyrin-5 [25], [26], Vps3 [27], Vps8 [27], POT1 [28] and caveolin [29], [30] have been identified. Identification of Rab5-interacting proteins has provided insights into the molecular mechanism of endocytosis. We recently identified plastins as Rab5-binding proteins and shows that these proteins are not only actin-binding proteins but also endocytosis regulators [31].

Vinculin is a 116-kDa cytoskeletal protein that is involved in the linkage of integrin adhesion molecules to the actin cytoskeleton [32]. Vinculin interacts with many proteins including talin [33], [34], [35], alpha-actinin [36], F-actin [37], [38], ARP2/3 [39], catenin [40], [41], [42], [43], Paxillin [44], Hic-5 [44], VASP [45] and vinexin [46], [47], [48]. Structurally, vinculin is divided into three main domains: an N-terminal head, a flexible proline-rich hinge (neck) region, and a C-terminal tail domain [49]. The protein's activity is regulated by conformational reorganization of these domains. Intramolecular associations between the head and tail domains constrain vinculin in an inactive conformation, causing it to be located within the cytoplasm [49]. The structure of vinculin can change to an open state (active conformation) that facilitates its localization to the plasma membrane [35], [50]. Many researchers have used various vinculin mutants to study the function of vinculin. Vinculin 8/19 (vin8/19) and vinculin T12 (vinT12) mutants interfere with the head–tail interaction characterizing constitutive activation [51]. The vinculin A50I (vinA50I) mutant inhibits the head/tail dissociation of vinculin [52] and increases the vinculin turnover rate in focal adhesions [53]. Vinculin can bind to phosphatidylinositol 4,5-biphosphate (PIP2) and has two lipid-binding sites: CT and H3 [50], [54]. The vinculin CT (vinCT) mutant was mutated in the CT lipid-binding site, the vinculin H3 (vinH3) mutant was mutated in the H3 lipid-binding site, and the vinculin LD (vinLD) mutant was mutated in both the CT and H3 lipid-binding sites. These mutants were deficient in PIP2. In addition, the vinculin D1 domain (residues 1-258) bound to talin and a-actinin by using vinculin deletion mutants [32]. A previous study showed by using collagen-coated beads that vinculin may be involved in cellular [55]. However, molecular mechanism of endocytosis by vinculin is still not clear.

Cellular responses to many external stimuli involve the activation of several types of MAPK signaling pathways. MAPKs are a family of serine/threonine kinases that comprise three major subgroups: ERKs, p38, and JNKs [56], [57]. MAPKs can be localized to early endosomes by various external stimuli via endocytosis, thereby transmitting signals to downstream [58], [59], [60], [61]. These proteins regulate the expression of many inflammatory cytokines including IL-6 [56], [57].

*Staphylococcus aureus* (*S. aureus*), a gram-positive pathogen, has long been recognized as one of the most important bacteria that cause various diseases such as bloodstream infections, bone and joint infections, and pneumonia. *In vitro* studies have shown that *S. aureus* is internalized [62] and survives inside non-phagocytic cells [63], [64], [65]. Internalized *S. aureus* is able to evade or delay elimination by the host's immune system and avoid extracellular antibiotics [66]. However, the invasive mechanism and the effect of *S. aureus* on host cells remain a mystery. In this study, we showed the role of vinculin–Rab5 interaction in the uptake of *S. aureus* into non-phagocytic cells and the relationship between these proteins and phosphorylation of MAPK and IL-6.

## Materials and Methods

### Ethics statement

This study was carried out in strict accordance with the recommendations in the Guide for the Care and Use of Laboratory Animals of the National Institutes of Health. The protocol was approved by the Committee on the Ethics of Animal Experiments of Tokyo Dental College.

### Cell culture

Cos-7 cells (RIKEN BIORESOURCE CENTER) were cultured in DMEM (Wako) supplemented with 10% FBS, 1% penicillin, and 1% streptomycin. HeLa cells (RIKEN BIORESOURCE CENTER) cells were cultured in Advanced MEM (Sigma) supplemented with 5% FBS, 1% penicillin, and 1% streptomycin.

### Antibodies

Antibodies were obtained from the following sources: anti-mouse HA and anti-rabbit HA (Sigma); anti-rabbit IgG-Alexa 555 and anti-rabbit IgG-Alexa 633 (Invitrogen); anti-rabbit Rab5, anti-mouse GFP, and anti-rabbit GFP (Novus); anti-mouse p38, anti-mouse JNK, and anti-mouse Erk (BD bioscience); anti-rabbit phospho-p38, anti-rabbit phospho-JNK, and anti-rabbit phospho-Erk (Cell Signaling Technology); anti-mouse vinculin, anti-IL-6, and anti-rabbit *S. aureus* (Abcam); anti-mouse IgG-HRP and anti-rabbit IgG-HRP (IBL); anti-GST HRP conjugate (Amersham Bioscience); anti-mouse GAPDH (MBL); and anti-mouse His (Sino Biological).

### Vector constructs

GFP-Rab5 (WT: wild type), GFP-Rab5S34N, and GFP-Rab5Q79L in pcDNA3 and GST-Rab5Q79L and GST-Rab5S34N in pGEX-2T constructs were kindly provided by Dr. Y. Yamamoto (Tokyo University of Agriculture, Tokyo, Japan). For the expression of HA-fused proteins, Rab5Q79L, Rab5 (WT), and Rab5S34N DNAs were amplified by PCR and cloned into pCMV-HA. The GST-R5BD vector was kindly donated by Dr. G. Li (University of Oklahoma Health Science Center, Oklahoma City, USA). GFP-vinculin (GFP-vinWT), GFP-vinculin8/19 (GFP-vin8/19), and GFP-vinculinT12 (GFP-vinT12) vectors were kindly provided by Dr. S. W. Craig (The Johns Hopkins School of Medicine, Baltimore, USA). The pTag RFP-vinculin vector was obtained from Evrogen Inc. vin1-258, vin1-880, vin258-880, vin881-1066, and vin1-1066 (vinWT) were amplified by PCR and cloned into the vector pet30a or pcDNA3-GFP. GFP-vinculinA50I (GFP-vinA50I) was constructed by mutating wild-type vinculin using a QuikChange Site-Directed Mutagenesis Kit (STRATAGEN) according to the manufacturer's instructions.

### Expression in *Escherichia coli* and purification of proteins

GST-Rab5Q79L, GST-Rab5S34N, GST, and GST-R5BD were expressed in BL21-Codon Plus and purified as described previously [29], [67], [68], [69]. His-vin1-258, His-1-880, His258-880, His881-1066, and His-vin1-1066 (full length) were expressed in BL21-Codon Plus and purified with His Mag sepharose Ni (GE Healthcare) according to the manufacturer's instructions.

### Immunoprecipitation

To analyze the binding of vinculin and Rab5, cells were transfected with the indicated plasmids and lysed for 30 min at 4°C with a buffer (10 mM Tris, pH 7.6, 150 mM NaCl, 5 mM MgCl_2_, 1% NP-40, 0.5 µg/mL leupeptin, 2 µg/mL aprotinin, and 10 µg/mL PMSF). The clarified lysates were incubated with antibodies for 2 h at 4°C. The immune complexes were precipitated with protein A–Sepharose (Millipore) for 2 h at 4°C and then washed extensively with lysis buffer. The beads were resuspended in SDS sample buffer and assayed by western blotting.

### GST-Rab5 pull-down assays

Five μg GST-Rab5Q79L or GST-Rab5S34N was added to 40 µL of glutathione–Sepharose resin and incubated for 1 h at 4°C. The beads were washed with a wash buffer (20 mM HEPES, 100 mM NaCl, 5 mM MgCl_2_, and 1 mM dithiothreitol, pH 7.6), incubated with the cell lysate or purified His-vinculin for 60 min at 4°C, washed three times with the wash buffer, resuspended in an SDS sample buffer (62.5 mM Tris pH 6.8, 2% SDS, 10% glycerol and 100 mM 2-mercaptoethanol, 0.005% BPB), and analyzed by western blotting.

### Uptake assay

To measure the uptake of transferrin, albumin, and Lucifer yellow, cells were pre-incubated with serum-free DMEM without phenol red for 1 h at 37°C in 24-well plates and then incubated with 50 µg/mL transferrin Alexa Fluor 555 (Invitrogen), 50 µg/mL albumin Alexa Fluor 555 (Invitrogen), or 1 mg/mL Lucifer yellow lithium salt (Sigma) diluted with serum-free DMEM without phenol red for 2 h at 37°C or 4°C to measure the background level of uptake (negative control). After incubation, the cells were collected with ice-cold PBS, washed eight times with ice-cold PBS, lysed with PBS containing 1% Triton X-100, and centrifuged at 10,000×*g* for 20 min at 4°C. The signal intensity of the supernatant was measured using SpectraMax M3 (Molecular Devices).

To measure FM4-64 uptake, cells were pre-incubated with serum-free DMEM without phenol red for 1 h at 37°C in 96-well plates. Subsequently, 100 µg/mL FM4-64 (Invitrogen) diluted with serum-free DMEM without phenol red was added to the cells. Immediately, fluorescence from extracellular FM4-64 was analyzed using SpectraMax M3 to measure the background level of uptake. The cells were then incubated for 2 h at 37°C, and signal intensity was measured using SpectraMax M3 (Molecular Devices).

To measure *S. aureus* uptake, the cells were pre-incubated with serum-free DMEM without phenol red for 1 h at 37°C in 96-well plates. Subsequently, 1 mg/mL pHrodo red-labeled *S. aureus* BioParticles (Invitrogen) diluted with serum-free DMEM without phenol red was added to the cells. The cells were incubated for 2 h at 37°C, and signal intensity was measured using SpectraMax M3 (Molecular Devices).

### Immunostaining

Cells were fixed with 4% formaldehyde in PBS for 10 min and washed with PBS. Nonspecific binding of antibodies was blocked by incubation with 5% sheep serum in TBS-T for 60 min, followed by washing with TBS-T. The cells were incubated with a primary antibody in TBS-T for 60 min and washed with PBS. Bound primary antibodies were visualized with a secondary antibody in buffer A (10 mM Tris, pH 7.6, 300 mM NaCl, and 0.5% Tween 20). After extensive washing with buffer A, cells were mounted on slide glasses. The cells were observed using a confocal fluorescence microscope (Carl Zeiss Co., Ltd).

### Over-expression of proteins in cultured cells

Each DNA plasmid was transfected with Lipofectamine 2000 (Invitrogen) according to the manufacturer's instructions.

### Knockdown in cultured cells

Rab5 siRNA (catalog no. RAB5A-HSS108976), vinculin siRNA (catalog no. VCL-HSS111259), Stealth RNAi Negative Control Low GC Duplex, and Stealth RNAi Negative Control Medium GC Duplex were obtained from Invitrogen. These siRNAs were transfected with Lipofectamine 2000 (Invitrogen) according to the manufacturer's instructions..

### GST-R5BD pull-down assay

One hundred μg/mL *S. aureus* was added to the medium of cells transfected with GFP-Rab5 and incubated for indicated times at 37°C. The GST-R5BD pull-down assay was then performed as described previously [67], [70], [71] to measure the Rab5-GTP level. In brief, the cells were washed two times with PBS and lysed for 5 min in 1 mL lysis buffer (25 mM HEPES, pH 7.4, 100 mM NaCl, 5 mM MgCl_2_, 0.1% NP-40, 2% glycerol, 1 mM DTT, 0.5 µg/mL leupeptin, 2 µg/mL aprotinin, and 10 µg/mL PMSF). Lysis extracts were clarified by centrifugation at 10,000×*g* for 5 min at 4°C, and supernatants were incubated with 20 µL of GST-R5BD bound to glutathione–Sepharose 4B beads for 20 min at 4°C with slow stirring. The beads were subsequently washed with lysis buffer, re-suspended in SDS sample buffer, and assayed by western blotting.

### Effect of *S. aureus* on vinculin–Rab5 binding

Cells were pre-incubated with DMEM for 1 h at 37°C in 6-well plates. After pre-incubation, 100 µg/mL *S. aureus* (Invitrogen) was added to tissue culture cells, followed by incubation for the indicated time. The cells were then washed with PBS and lysed with lysis buffer (25 mM HEPES, pH 7.4, 100 mM NaCl, 5 mM MgCl_2_, 0.1% NP-40, 2% glycerol, and 1 mM DTT) containing protease inhibitors. The lysates were subjected to immunoprecipitation or the GST-R5BD pull-down assay as described earlier.

### Bacterial strain

*S. aureus* 209P was preserved at Tokyo Dental College. *S. aureus* 209P was grown in trypticase soy broth medium.

### Animals

Specific pathogen-free BALB/c female mice (age, 10 weeks) were obtained from Sankyo Labo Service Corporation, Inc., Tokyo, Japan. All mice were given sterile food and water *ad libitum* under conventional conditions in the animal care facility of Tokyo Dental College. This study was carried out in accordance with “Guidelines for the Treatment of Experimental Animals in Tokyo Dental College” [72].

### Knockdown *in vivo*

*In vivo* grade siRNA for Rab5 (catalog no. RAB5A-MSS212350), siRNA for vinculin (catalog no. VCL-MSS241137), Stealth RNAi Negative Control Low GC Duplex, and Stealth RNAi Negative Control Medium GC Duplex were obtained from Invitrogen. Two hundred fifty μL of 3 mg/mL siRNA was diluted to 1.5 mg/mL with 250 µL complexation buffer accessory for invivofectamine (Invitrogen). Five hundred µL of the diluted siRNA solution was mixed with 500 µL invivofectamine, and incubated for 30 min at 50°C. Forty μL of the mixture was injected into murine lungs via direct transtracheal instillation (30 µg siRNA per mouse). Note that Rab5 and vinculin knockdown continues for more than week (data not shown).

### Infection of *S. aureus* in murine lungs

Ten-week-old BALB/c female mice were infected with bacteria via direct transtracheal instillation with minor modifications [72]. The mice were anesthetized by injection of thiopental sodium (Ravonal, Tanabe-Mitsubishi, Osaka, Japan), and a surgical incision was made in the neck to expose the trachea. Approximately 2×10^7^ cfu of *S. aureus* 209P diluted in 50 µl PBS was instilled into the trachea using a 30-gauge needle. The incision was closed with a suture, and the mice were monitored over the next 3 days. The mice were sacrificed, and bronchoalveolar lavage (BAL) was performed three times with 1 mL sterile PBS. The lung was then removed and homogenized in sterile PBS, and diluted homogenate was plated onto trypticase soy agar plates. The plates were cultured at 37°C, after which colonies were counted as cfu.

### Statistics

Data were compiled and analyzed using Ezanova software. Statistical significance was defined as *p*<0.05.

## Results

### Vinculin binding with Rab5

**Figure 1:**
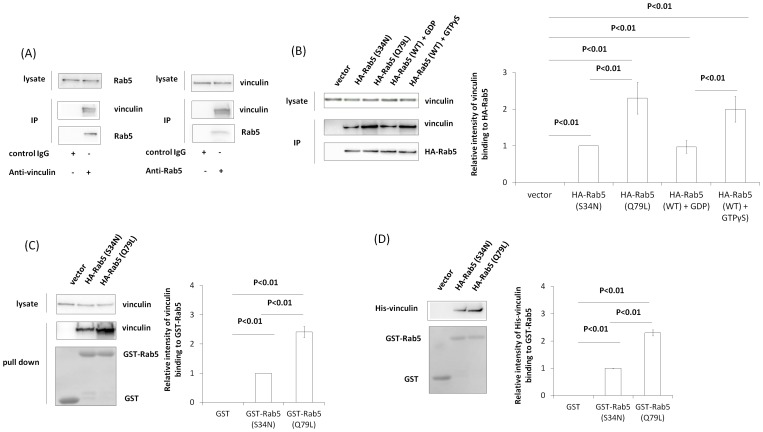
Direct binding of vinculin and Rab5. (A) Endogenous vinculin and Rab5 were immunoprecipitated from Cos-7 lysates with anti-Rab5 or anti-vinculin antibodies. Vinculin and Rab5 were assayed by western blotting using specific antibodies to vinculin and Rab5. (B) HA-Rab5 (S34N), HA-Rab5 (Q79L) and HA-Rab5 (WT) were transiently expressed in Cos-7 cells and subjected to immunoprecipitation with anti-HA antibody. Note that immunoprecipitation of HA-Rab5 (WT) was carried out with GTPγS and GDP. Proteins were assayed by western blotting. The graph shows mean ± S.E. values of three independent experiments (C) A pull-down assay from a Cos-7 lysate was performed using GST, GST-Rab5 (S34N), and GST-Rab5 (Q79L). Vinculin bound to the beads was assayed by western blotting, and GST, GST-Rab5 (S34N), and GST-Rab5 (Q79L) on a PVDF membrane were stained with Ponceau S. The graph shows mean ± S.E. values of three independent experiments (D) GST, GST-Rab5 (S34N), and GST-Rab5 (Q79L) were incubated with purified His-vinculin, and a pull-down assay was performed using glutathione beads. His-Vinculin bound to the beads was assayed by western blotting, and GST, GST-Rab5 (S34N), and GST-Rab5 (Q79L) on a PVDF membrane were stained with Ponceau S. The graph shows mean ± S.E. values of three independent experiments.

To investigate whether vinculin can bind to Rab5, we performed immunoprecipitation using Cos-7 lysates. Endogenous vinculin and Rab5 were coimmunoprecipitated with both anti-vinculin and anti-Rab5 for immunoprecipitation (Fig. 1A). We then investigated whether vinculin interacts with inactive Rab5 and/or active Rab5. HA-Rab5 (Q79L) (activated mutant), HA-Rab5 (S34N) (inactivated mutant) and HA-Rab5 (WT) were overexpressed in Cos-7 cells and immunoprecipitated using anti-HA antibody. Immunoprecipitation showed that vinculin bound to both HA-Rab5 (Q79L) and HA-Rab5 (S34N), but bound more strongly to HA-Rab5 (Q79L) than to HA-Rab5 (S34N) (Fig. 1B). It is also showed that vinculin bound to both HA-Rab5 (WT)-GTPγS and HA-Rab5 (WT)-GDP, but bound more strongly to HA-Rab5 (WT)-GTPγS than to HA-Rab5 (WT)-GDP (Fig. 1B). We next tested vinculin–Rab5 interaction by the GST pull-down assay. GST pull-down assays using Cos-7 cell lysates also showed that vinculin bound to both GST-Rab5 (Q79L) and GST-Rab5 (S34N) but bound more strongly to GST-Rab5 (Q79L) than to GST-Rab5 (S34N) (Fig. 1C). Purified His-vinculin also bound strongly to GST-Rab5 (Q79L) (Fig. 1D). Together, these results indicate that vinculin can interact directly with Rab5 *in vitro*.

### Effect of vinculin and Rab5 on uptake

**Figure 2:**
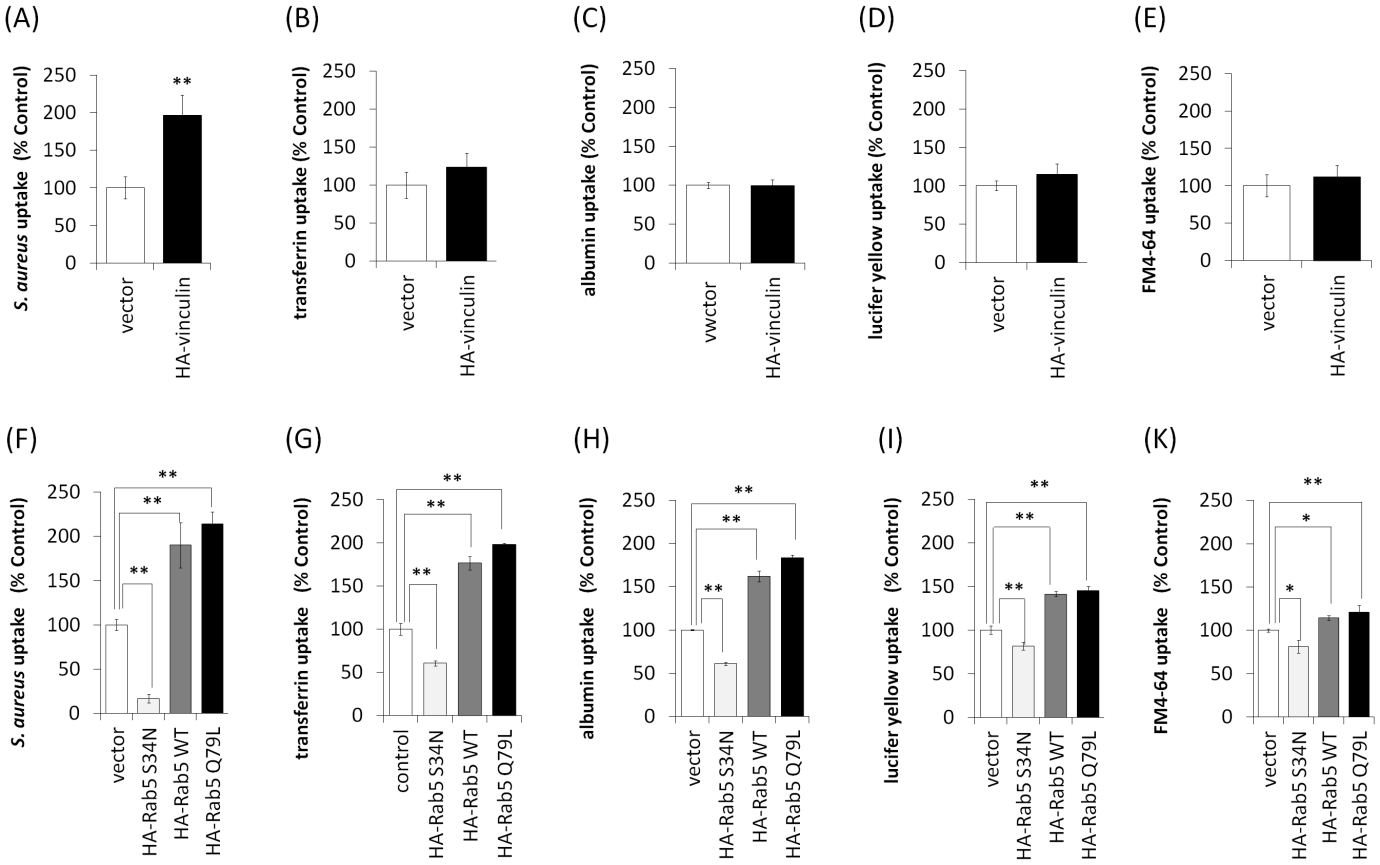
Effect of vinculin and Rab5 expression on cellular uptake. (A–E) HA-vinculin-expressing Cos-7 cells were incubated with various markers of uptake at 37°C for 2 h. Uptake of pHrodo red-labeled *S. aureus* BioParticles increased in HA-vinculin-expressing cells, but the uptake of transferrin Alexa 555, albumin Alexa 555, Lucifer yellow and FM 4-64 did not increase. Error bar: n = 3−6±SE, **p<0.01. (F–H) HA-Rab5 (S34N)-, HA-Rab5 (WT)-, and HA-Rab5 (Q79L)-expressing Cos-7 cells were incubated with various uptake indicators at 37°C for 2 h. Uptake of pHrodo red-labeled *S. aureus*, transferrin Alexa 555, albumin Alexa 555, Lucifer yellow, and FM 4-64 increased in HA-Rab5 (WT)- and HA-Rab5 (Q79L)-expressing cells, whereas uptake of each of the indicators was decreased in HA-Rab5 (S34N)-expressing cells. Error bar: n = 3–6±SE, *p<0.05, **p<0.01.

**Figure 3:**
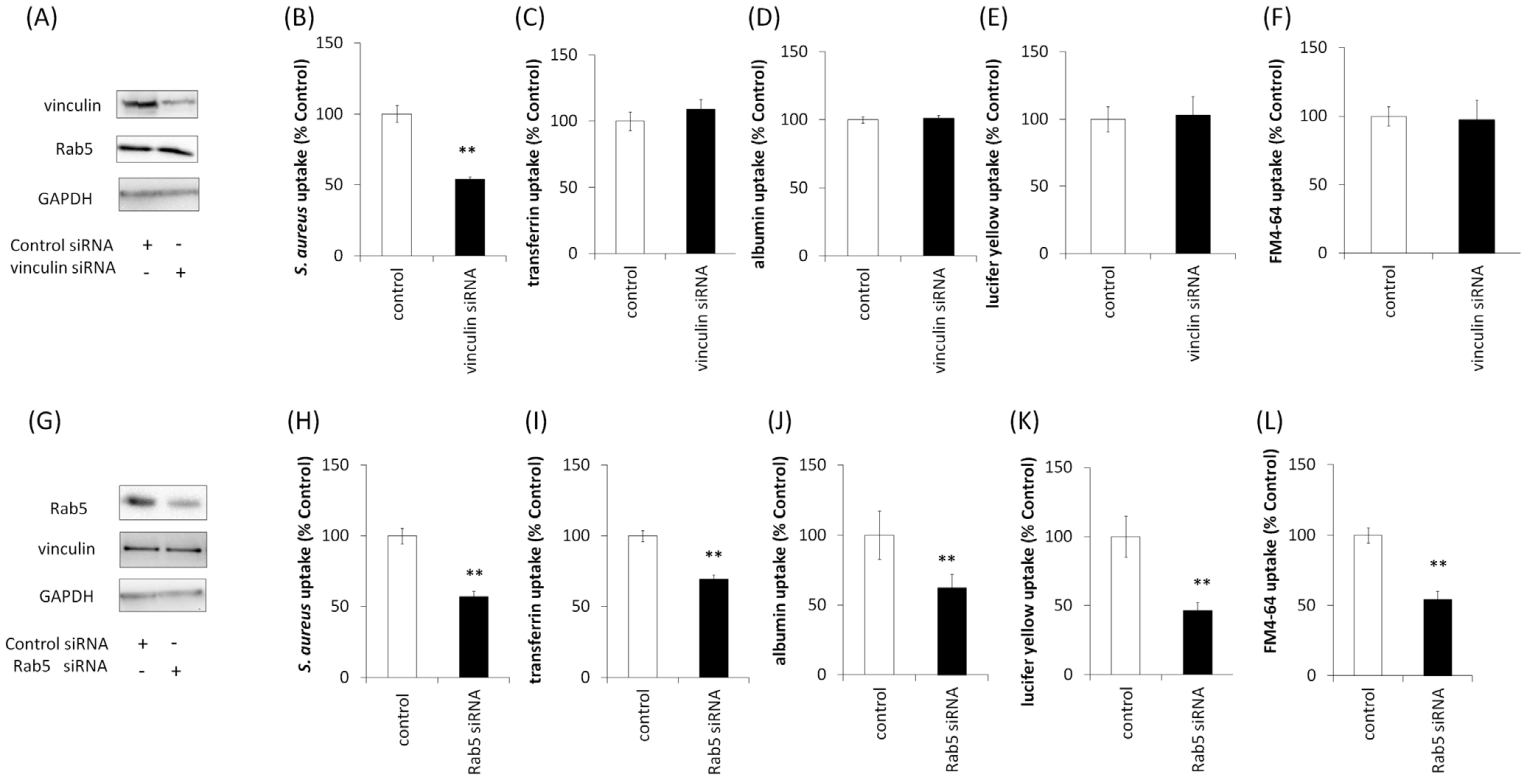
Effects of vinculin and Rab5 knockdown on cellular uptake. (A) siRNAs for vinculin were transfected into HeLa cells, and cell lysates were assayed by western blotting using anti-vinculin, anti-Rab5, and anti-GAPDH antibodies (internal control). (B–F) Various uptake indicators were added to the media of vinculin knockdown cells and incubated at 37°C for 2 h. Uptake of pHrodo red-labeled *S. aureus* decreased, but that of transferrin Alexa 555, albumin Alexa 555, Lucifer yellow, and FM 4-64 did not decrease. Error bar: n = 3−6±SE, **p<0.01. (G) siRNAs for Rab5 were transfected into HeLa cells and cell lysates were assayed by western blotting with anti-Rab5, anti-vinculin, and anti-GAPDH antibodies. (H–L) HeLa cells transfected with Rab5 siRNA. Various uptake indicators were added to the media of Rab5 knockdown cells and incubated at 37°C for 2 h. Uptake of pHrodo red-labeled *S. aureus*, transferrin Alexa 555, albumin Alexa 555, Lucifer yellow, and FM 4-64 was decreased. Error bar: n = 3−6±SE, **p<0.01.

We hypothesized that vinculin plays a role in cellular uptake, given that Rab5 is important for endocytosis. We performed uptake assays using various markers. HA-vinculin over-expression increased the uptake of *S. aureus* (Fig. 2A) but not that of transferrin, albumin, Lucifer yellow, or FM4-64 in Cos-7 cells (Fig. 2B–E). In contrast, HA-Rab5Q79L and HA-Rab5 (WT) over-expression increased the uptake of *S. aureus*, transferrin, albumin, Lucifer yellow, and FM4-64, whereas HA-Rab5S34N over-expression decreased their uptake (Fig. 2F–K). To further investigate the effect of vinculin and Rab5 knockdown on uptake, we performed knockdown with specific siRNAs. As shown in Fig. 3A, introducing vinculin siRNA into HeLa cells inhibited vinculin expression. Vinculin knockdown decreased the uptake of *S. aureus* in HeLa cells (Fig. 3B) but not that of transferrin, albumin, Lucifer yellow, or FM4-64 (Fig. 3C–F). As shown in Fig. 3G, introducing Rab5 siRNA into HeLa cells inhibited Rab5 expression. With Rab5 knockdown, uptake of *S. aureus*, transferrin, albumin, Lucifer yellow, and FM4-64 was inhibited in HeLa cells (Fig. 3H–L). These results suggested that Rab5 is involved in the uptake of various markers, whereas vinculin is involved in only *S. aureus* uptake.

### Effects of *S. aureus* on vinculin-Rab5 binding and Rab5-GTP

**Figure 4:**
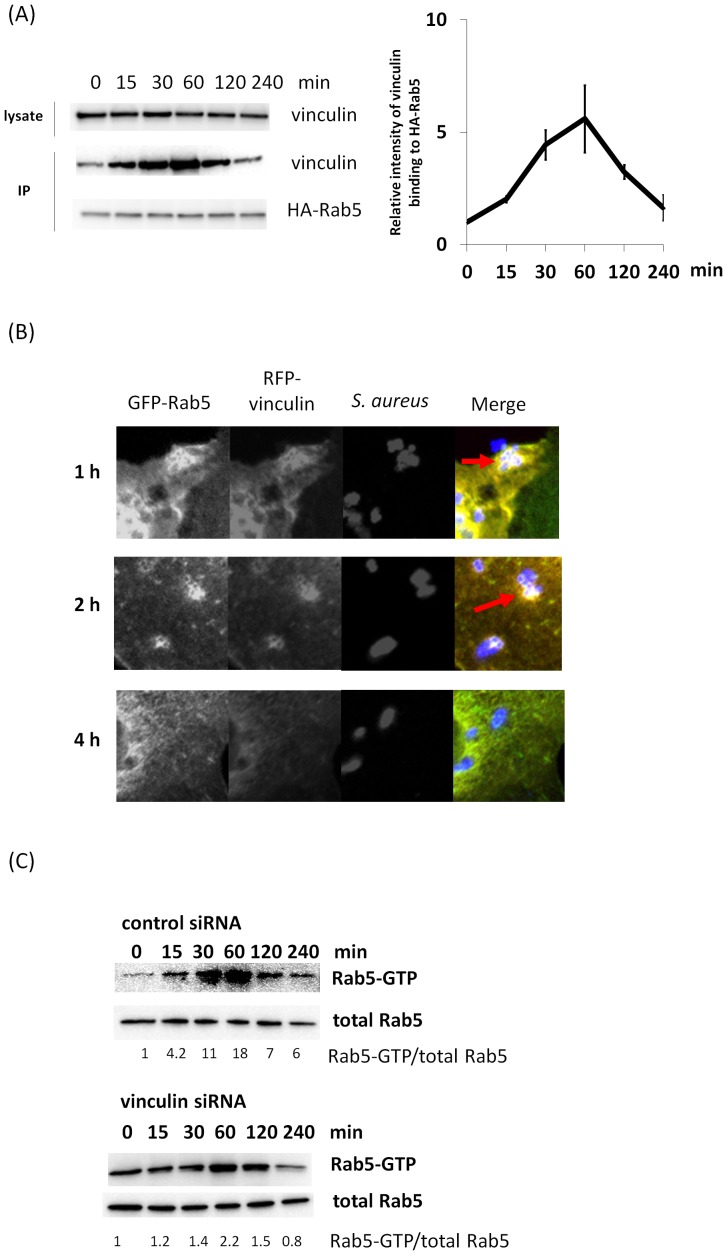
Effects of *S. aureus* on vinculin-Rab5 binding and Rab5-GTP. (A) S. aureus was added to the medium of HA-Rab5 (WT)-transfected Cos-7 cells and incubated with *S. aureus* for the indicated time at 37°C. The cells were lysed and subjected to immunoprecipitation with anti-HA antibody. Immunocomplexes were assayed by western blotting with anti-HA and anti-vinculin antibodies. Proteins levels were quantified using ImageJ in three independent experiments. (B) *S. aureus* was added to the medium of Cos-7 cells expressing GFP-Rab5 (WT) and RFP-vinculin (WT) and incubated for the indicated time at 37°C. The cells were fixed and immunostained with *S. aureus* antibodies. (C) HeLa cells were transfected with GFP-Rab5 (WT) and vinculin siRNA or control siRNA. *S. aureus* was added to the medium of transfected cell and incubated for the indicated time at 37°C. The cells were lysed and subjected to a GST-R5BD pull-down assay. GST-R5BD-bound beads and lysates were assayed by western blotting with anti-GFP antibody. Rab5-GTP levels were normalized to total GFP-Rab5 levels and quantified using ImageJ.

Since both vinculin and Rab5 were shown to be involved in *S. aureus* uptake (Fig 2A and F and Fig. 3B and H), we next investigated the effect of *S. aureus* on vinculin–Rab5 interaction. *S. aureus* was added to HA-Rab5 (WT)-expressing Cos-7 cells, and the cell lysate was immunoprecipitated with anti-HA antibody. As shown in Fig. 4A, vinculin–Rab5 interaction increased up to 60 min following the addition of *S. aureus*. We further examined the effect of *S. aureus* on vinculin–Rab5 interaction with confocal fluorescence microscopy. *S. aureus* was added to GFP-Rab5 (WT) and RFP-vinculin-expressing Cos-7 cells, and the cells were immunostained with anti-*S. aureus* . Confocal fluorescence microscopic analysis showed that RFP–vinculin colocalized with GFP-Rab5 on *S. aureus* positive endosomes in Cos-7 cells at 1 and 2 h (Fig. 4B), whereas RFP-vinculin did not colocalize with GFP-Rab5 at 4 h. These findings indicate that *S. aureus* is involved in the vinculin-Rab5 interaction in the early stage of endocytosis.

We then performed a GST-R5BD pull-down assay [67] to investigate Rab5 activation in cultured cells. To investigated the effect of vinculin on Rab5 activation in *S. aureus* uptake, *S. aureus* was added to HeLa cells showing vinculin knockdown and overexpressing GFP-Rab5 (WT), and the cell lysate was analyzed using a GST-R5BD pull-down assay. In contrast to the control cells, introduction of vinculin siRNA decreased the level of Rab5-GTP with the addition of *S. aureus* (Fig. 4C). These findings suggest that vinculin is involved in Rab5 activity in *S. aureus* uptake.

### Effect of active vinculin mutants on *S. aureus* uptake

**Figure 5:**
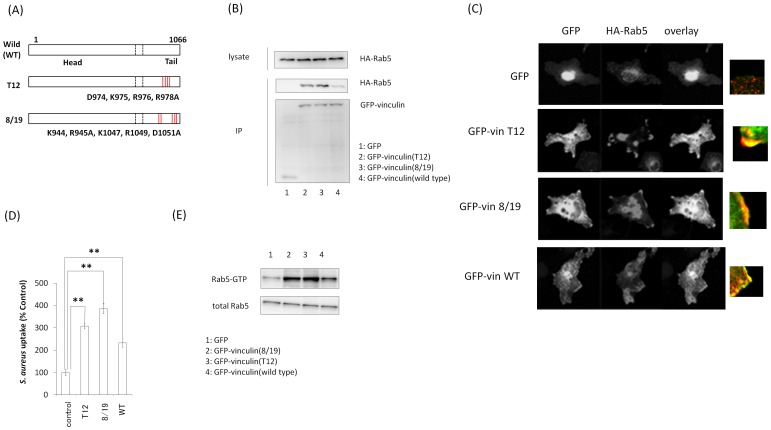
Effect of vinculin-activated mutants on *S. aureus* uptake. (A) Schematic of active vinculin mutants. (B) GFP, GFP-Vin8/19, GFP-VinT12, and GFP-vinculin (WT) were coexpressed with HA-Rab5 (WT) in Cos-7 cells, and these proteins were immunoprecipitated with anti-GFP antibody. Immunocomplexes were assayed by western blotting with anti-GFP and anti-HA antibodies. (C) GFP, GFP-Vin8/19, GFP-VinT12, and GFP-vinculin (WT) were coexpressed with HA-Rab5 (WT) in Cos-7 cells and immunostained with anti-HA antibody. (D) pHrodo red-labeled *S. aureus* was added to the medium of Cos-7 cells expressing active vinculin mutants and incubated for 2 h at 37°C. The graph shows mean ± S.E. values of six independent experiments, **p<0.01. (E) Cos-7 cells were transfected with HA-Rab5 (WT) and active vinculin mutants. *S. aureus* was added to the medium of transfected cells and incubated for 60 min at 37°C. The cells were lysed and subjected to a GST-R5BD pull-down assay. GST-R5BD-bound beads and lysates were assayed by western blotting with anti-HA antibody.

We then investigated whether vinculin activation is involved in *S. aureus* uptake. Vinculin 8/19 (vin8/19) and vinculin T12 (vinT12) (Fig. 5A) interfere with the head–tail interaction characterizing constitutive activation [51]. Immunoprecipitation assays showed that GFP-vin8/19 and T12 strongly bound to Rab5 (Fig. 5B). Confocal fluorescence microscopic analysis revealed that GFP-vin8/19, GFP-vinT12 mutants and GFP-vin (WT) change the localization of HA-Rab5 and that the vinculin mutants and vin (WT) were colocalized with HA-Rab5 (WT) close to the plasma membrane. (Fig. 5C). Moreover, vin8/19 and T12 mutants strongly facilitated *S. aureus* uptake in cells. (Fig. 5D). We further investigated the effect of active vinculin mutants on Rab5 activation in *S. aureus* uptake using GST-R5BD. *S. aureus* was added to Cos-7 cells showing over-expression of active vinculin mutants and HA-Rab5 (WT), and the cell lysate was analyzed using a GST-R5BD pull-down assay. Over-expression of active vinculin mutants increased the level of Rab5-GTP with the addition of *S. aureus* (Fig. 5E). These findings suggest that vinculin activation is important for *S. aureus* uptake.

### Effect of inactive vinculin mutants on *S. aureus* uptake

**Figure 6:**
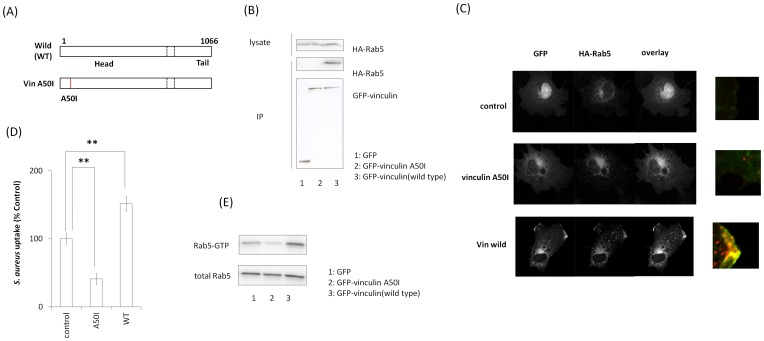
Effect of vinculin-inactivated mutants on *S. aureus* uptake. (A) Schematic of inactive vinculin mutants. (B) GFP, GFP-VinA50I, and GFP-vinculin (WT) were coexpressed with HA-Rab5 (WT) in Cos-7 cells, and these proteins were immunoprecipitated with anti-GFP antibody. Immunocomplexes were assayed by western blotting with anti-GFP and anti-HA antibodies. (C) GFP, GFP-VinA50I, and GFP-vinculin (WT) were coexpressed with HA-Rab5 (WT) in Cos-7 cells and immunostained with anti-HA antibody. (D) pHrodo red-labeled *S. aureus* was added to the medium of Cos-7 cells expressing inactive vinculin mutants and incubated for 2 h at 37°C. The graph shows mean ± S.E. values of six independent experiments, **p<0.01. (E) Cos-7 cells were transfected with HA-Rab5 (WT) and inactive vinculin mutants. *S. aureus* was added to the medium of transfected cells and incubated for 60 min at 37°C. The cells were lysed and subjected to a GST-R5BD pull-down assay. GST-R5BD-bound beads and lysates were assayed by western blotting with anti-HA antibody.

Vinculin A50I (vinA50I) mutant (Fig. 6A) inhibits the head/tail dissociation of vinculin characterizing constitutive inactivation [52]. Immunoprecipitation assays showed that GFP-vinA50I did not bind to HA-Rab5 (WT) (Fig. 6B). Confocal fluorescence microscopic analysis revealed that vinA50I did not colocalize with HA-Rab5 (WT) close to the plasma membrane (Fig. 6C). Moreover, vinA50I suppressed *S. aureus* uptake. (Fig. 6D). We further investigated the effect of the inactive vinculin mutant on Rab5 activation in *S. aureus* uptake using GST-R5BD. *S. aureus* was added to Cos-7 cells showing over-expression of the inactive vinculin mutant and HA-Rab5 (WT), and the cell lysate was analyzed using a GST-R5BD pull-down assay. Over-expression of the inactive vinculin mutant decreased the level of Rab5-GTP with the addition of *S. aureus* (Fig. 6E). These findings suggest that vinculin inactivation decreases *S. aureus* uptake.

### Functional analysis of the Rab5-binding domain of vinculin

**Figure 7:**
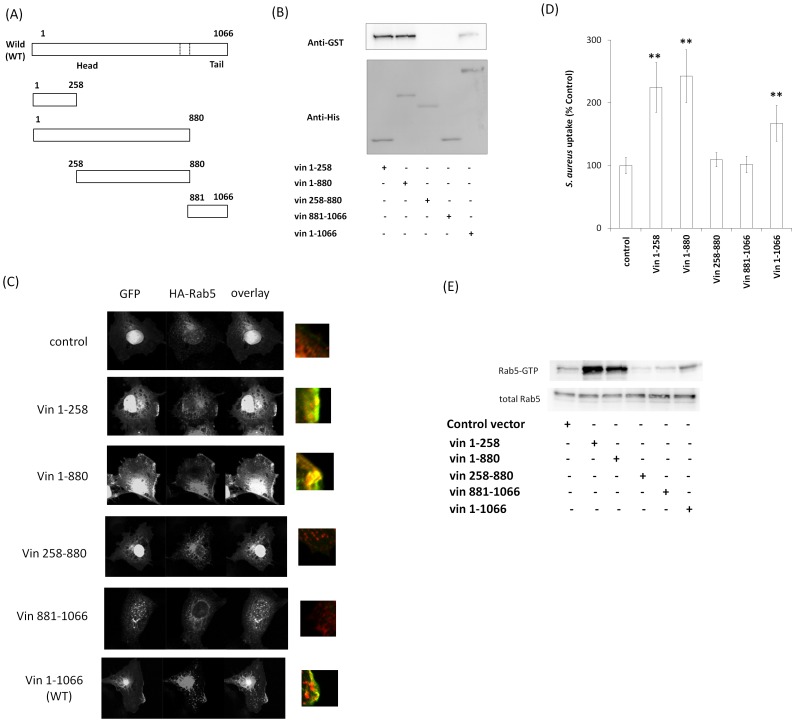
Determination of the Rab5-binding domain of vinculin. (A) Schematic of vinculin deletion mutants. (B) GST-Rab5 (Q79L) was incubated with purified His-vinculin deletion mutants, and a His-pull-down assay was performed. The beads were assayed by western blotting. (C) GFP, GFP-Vin1-258, GFP-Vin1-880, GFP-Vin258-880, GFP-Vin881-1066 and GFP-vinculin (full length) were coexpressed with HA-Rab5 in Cos-7 cells and immunostained with anti-HA antibody. (D) pHrodo red-labeled *S. aureus* was added to the medium of Cos-7 cells expressing each vinculin deletion mutant and incubated for 2 h at 37°C. The graph shows mean ± S.E. values of six independent experiments, **p<0.01. (E) Cos-7 cells were transfected with HA-Rab5 (WT) and vinculin deletion mutants. *S. aureus* was added to the medium of transfected cells and incubated for 60 min at 37°C. The cells were lysed and subjected to a GST-R5BD pull-down assay. GST-R5BD-bound beads and lysates were assayed by western blotting with anti-HA antibody.

Since vinculin directly bound to Rab5 (Fig. 1D), we next examined the Rab5-binding domain in vinculin. We constructed His-tagged deletion mutants of vinculin and examined the interaction with Rab5 by pull-down assays. As shown in Fig. 7A. His-vin1-258, His-vin1-880, and His-vin1-1066 (full length) bound to GST-Rab5 (Q79L), whereas vin258-880 and vin881-1066 did not (Fig. 7B). In addition, confocal fluorescence microscopic analysis showed that vin1-258, vin1-880, and vin1-1066 colocalized with HA-Rab5 (WT) close to the plasma membrane, but vin258-880 and vin881-1066 did not (Fig. 7C). These findings indicate that the N terminus of vinculin (vin1-258) can bind to Rab5. Next, we investigated the role of these vinculin mutants in *S. aureus* uptake. Cos-7 cells co-transfected with each of the vinculin mutants with HA-Rab5 were incubated with *S. aureus*. As shown in Fig. 7D, vin1-258, vin1-880, and vin1-1066 facilitated *S. aureus* uptake. Furthermore, Rab5-GTP induced by *S. aureus* was enhanced by over-expression of the vin1-258, vin1-880, and vin1-1066 in cos-7 cells (Fig. 7E). These findings suggest that N terminus of vinculin (vin1-258) is important for vinculin-Rab5 binding and *S. aureus* uptake.

### Decrease in *S. aureus*-induced phosphorylation of p38, Erk and JNK and decrease in IL-6 expression by vinculin and Rab5 knockdown

**Figure 8:**
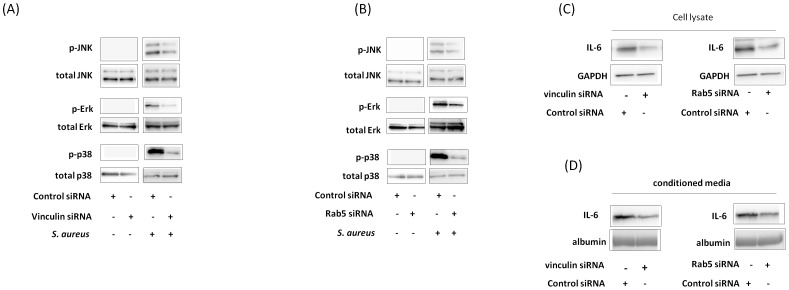
Effect of vinculin and Rab5 knockdown on *S. aureus*-induced IL-6 expression. (A and B) *S. aureus* was added to the medium of HeLa cells with vinculin (A) or Rab5 (B) knockdown and incubated for 60 min at 37°C. The cell lysates were assayed with anti-JNK, anti-phosphor-JNK, anti-ERK, anti-phosphor-Erk, anti-p38, and anti-phosphor-p38 antibodies by western blotting. (C and D) *S. aureus* was added to the medium of HeLa cells with vinculin or Rab5 knockdown and incubated for 16 h. The cell lysate (C) and conditioned medium (D) were assayed with anti-IL-6 antibody by western blotting. GAPDH and albumin were internal controls.

MAPK is stimulated by bacterial infection, leading to cytokine expression [73]. We accordingly investigated whether *S. aureus* affects IL-6 expression via MAPK. p38, Erk, and JNK phosphorylation was increased by *S. aureus*. With vinculin and Rab5 knockdown, *S. aureus*-induced p38, Erk, and JNK phosphorylation in the cells was decreased (Fig. 8A and B). With vinculin and Rab5 knockdown, *S. aureus*-induced IL-6 expression was decreased in the cell lysate and medium (Fig. 8C and D). These results suggest that vinculin and Rab5 are involved in *S. aureus*-induced phosphorylation of p38, Erk and JNK and *S. aureus*-induced IL-6 expression.

### Functional consequences of vinculin and Rab5 knockdown *in vivo*

**Figure 9:**
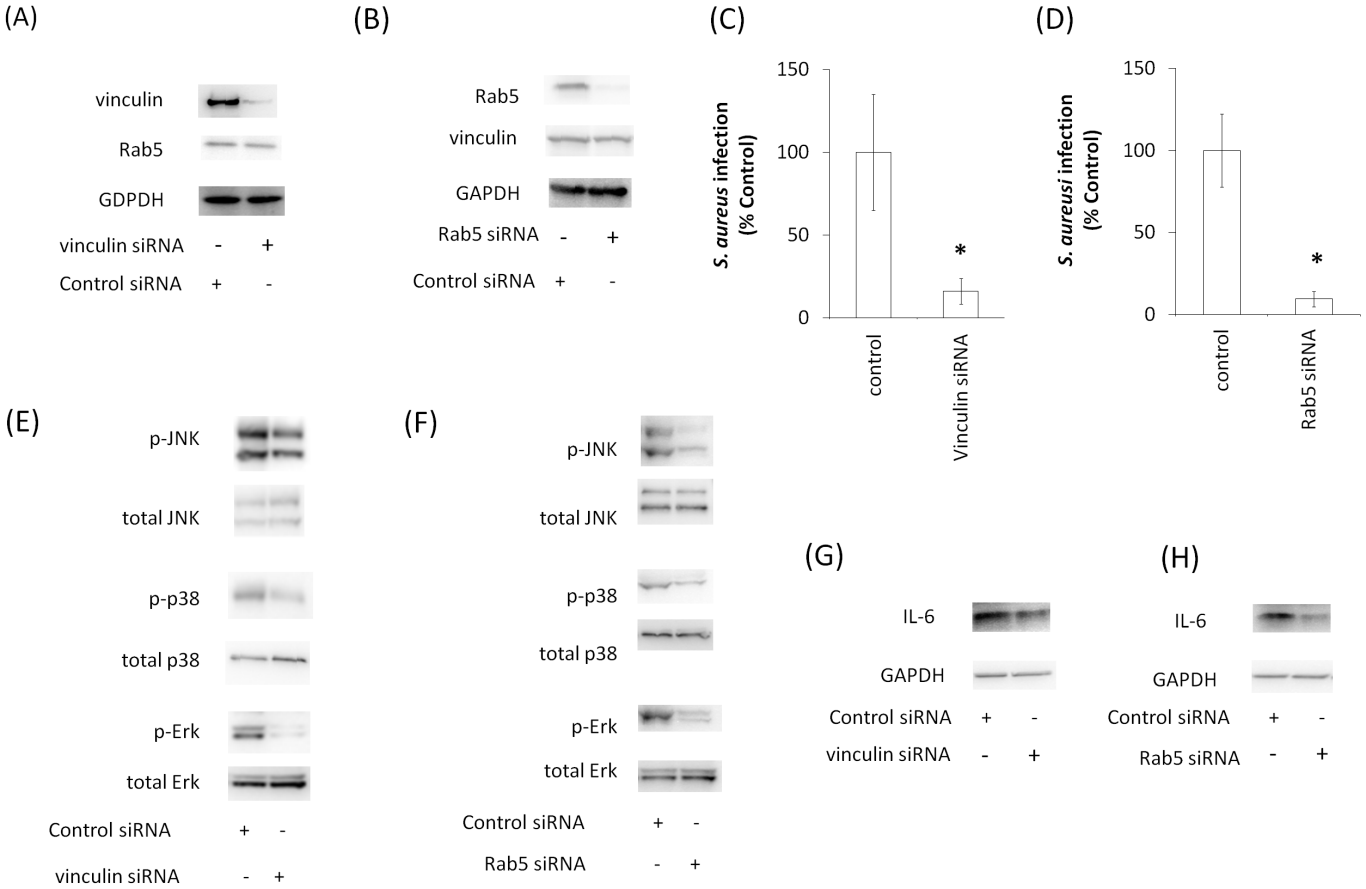
Effects of vinculin and Rab5 knockdown on *S. aureus* infection in murine lung. (A and B) Mouse lungs were transfected with vinculin and Rab5 siRNA. After 3 days, lungs were homogenized and treated with anti-vinculin, anti-Rab5, and anti-GAPDH (internal control) antibodies. (C and D) Murine lungs in which vinculin or Rab5 had been knocked down were infected with *S*. *aureus*. Lung tissues were analyzed for colony formation. The graph shows the mean ± S.E. of five independent experiments. (E–F) Murine lung in which vinculin (E and G) or Rab5 (F and H) had been knocked down was infected with *S*. *aureus*. The murine lung homogenates were assayed with anti-JNK, anti-phosphor-JNK, anti-ERK, anti-phosphor-Erk, anti-p38, anti-phosphor-p38, anti-IL-6, and anti-GAPDH (internal control) antibodies by western blotting.

To obtain evidence for the importance of vinculin and Rab5 *in vivo*, we introduced siRNA into the mouse lung. First, we confirmed the knockdown levels of vinculin and Rab5 in the mouse lung after introducing siRNAs for vinculin and Rab5. Western blotting showed that the expression levels of vinculin and Rab5 in the lung were reduced by these siRNAs (Fig. 9A and B). We next infected mouse lungs with *S*. *aureus* and analyzed lung tissue by colony formation assays. In contrast to the control, vinculin knockdown reduced infection by *S*. *aureus* in mouse lungs (Fig. 9C). When Rab5 was knocked down, infection by *S. aureus* was also reduced in mouse lungs (Fig. 9D). Furthermore, *S. aureus*-induced p38, Erk, and JNK phosphorylation in the mouse lung decreased with vinculin and Rab5 knockdown (Fig. 9E and F). Moreover, *S. aureus*-induced IL-6 expression in the mouse lung decreased with vinculin and Rab5 knockdown (Fig. 9G and H).

## Discussion

In this study, we demonstrated that vinculin interacts with Rab5 and modulates functions of the protein in manner different from those for other well-known Rab5-interacting proteins as follows. First, vinculin is a focal adhesion protein, unlike other Rab5-interacting proteins (Rabaptin-5, EEA1, and Rabex-5), which are recruited to early endosome membranes by active Rab5 [2], [20], [74]. Second, vinculin interacts with both inactive Rab5 and active Rab5, and this property also distinct from EEA-1, Rabex-5, and Rabaptin-5. Third, vinculin appears to participate in only phagocytosis (internalization of *S. aureus* into cells).

Recent findings suggest that vinculin may participate in the pahgocytosis of collagen beads [55]. In the present study, vinculin was shown to be involved in *S. aureus* uptake but not in the uptakes of transferrin, albumin, Lucifer yellow, or FM4-64 (Fig. 2A–E and Fig. 3B–F). Phagocytosis is a process by which cells engulf large particles, usually over 0.5 µm in diameter [75]. *S. aureus* is over 0.5 µm in diameter, and cells engulf *S. aureus* by phagocytosis via cell surface receptor such as integrins [76]. In contrast, the sizes of transferrin, albumin, Lucifer yellow and FM4-64 are very small and thus they do not induce phagocytosis. Transferrin is internalized to cells by clathrin-dependent endocytosis via the transferrin receptor [77]. Albumin is mainly incorporated into cells by caveolae-dependent endocytosis via gp60, an albumin receptor [78]. Lucifer yellow and FM4-64, which do not have specific receptors, are ingested by fluid-phase endocytosis [79], [80], [81]. It is likely that vinculin cannnot participate in receptor-mediated endocytosis of small molecules and fluid-phase endocytosis; however, vinculin may be a critical protein for endocytosis of large molecules such as those incorporated into cells by phagocytosis.

In the present study, vinculin was shown to bind to both inactive and active Rab5 (Fig. 1B–D) and to be involved in Rab5 activation during *S. aureus* uptake (Fig. 4C). Rab5 activation requires interaction with GEF, which contains a specific, highly conserved domain (Vps9) that catalyzes nucleotide exchange on Rab5 [20], [82], [83], [84], [85], [86], [87], [88], [89]. However, the amino acid sequence of vinculin does not contain a Vps9 domain. RabGDIs regulates the GDP/GTP exchange reaction of most Rab proteins by inhibiting the dissociation of GDP from them and the subsequent binding of GTP to them [4]. Although we have not uncovered the mechanism of vinculin-mediated Rab5 activation, vinculin may release RabGDI from Rab5-GDP or associate with GEF. Moreover, our data indicated that vinculin could bind more strongly to active Rab5 than to inactive Rab5, although vinculin bound to both of the molecules (Fig. 1B–D). Active Rab5 is crucial for vesicle transportation in the early stage of endocytosis (also phagocytosis) and early endosme fusion [2], [4]. It is possible that vinculin is involved in vesicle transport and/or endosome fusion in the early stage of phagocytosis. In support of this, we have observed that there are interactions between vinculin and other early endsome proteins such as EEA1 and Rabaptin-5 (our unpublished data).

Assays with active vinculin mutants (vinT12 and vin8/19) showed that vinculin activation facilitated vinculin–Rab5 binding and *S. aureus* uptake (Fig. 5B and D), whereas an inactive vinculin mutant (vinA50I) decreased *S. aureus* uptake (Fig. 6D). It was recently shown that active vinculin mutants recruit vinexin, a vinculin-interacting protein, to the plasma membrane [48]. It is possible that active vinculin also recruits Rab5 to the plasma membrane, thereby inducing phagocytosis of *S. aureus*.

*S. aureus*, a gram-positive pathogen, has long been recognized as one of the most important bacteria that cause various diseases including pneumonia [90]. *In vitro* studies have shown that *S. aureus* is internalized and survives inside non-phagocytic cells [62]. Internalized *S. aureus* is able to evade or delay elimination by the host's immune system and avoid extracellular antibiotics [66]. Therefore, a drug that inhibits internalization of *S. aureus* into non-phagocytic cells could be valuable for therapy of pneumonia. Our data showed that vinculin and Rab5 participated in infection of *S. aureus* in the mouse lung (Fig. 9C and D). Both vinculin and Rab5 knockdown also decreased *S. aureus*-induced IL-6 expression (Fig. 9E–H), indicating that inflammation was inhibited. Vinculin might be a target of therapy for various *S. aureus*-induced diseases including pneumonia.

In conclusion, we have shown that vinculin interacts directly with Rab5 and that its interaction is involved in *S. aureus* uptake. However, we did not observe uptake of other substances under the influence of vinculin–Rab5 interaction. Vinculin could interact with Rab5 without *S. aureus* (Fig. 1A–D). Vinculin underpins integrin under the plasma membrane, and integrin binds to extracellular matrix proteins, many bacteria, bacterial pathogens and viruses [91], [92], [93]. A recent study showed that R-Ras/Rin/Rab5 complex controls endothelial cell adhesion and morphogenesis via active integrin endocytosis [94]. To elucidate the precise molecular mechanisms, further studies are needed to provide new insights into the mechanisms of cellular uptake through vinculin–Rab5 interaction.
